# Host genetic polymorphisms and serological response against malaria in a selected population in Sri Lanka

**DOI:** 10.1186/s12936-018-2622-9

**Published:** 2018-12-17

**Authors:** Rajika L. Dewasurendra, Anna Jeffreys, Sharmini A. Gunawardena, Naduviladath V. Chandrasekharan, Kirk Rockett, Dominic Kwiatkowski, Nadira D. Karunaweera

**Affiliations:** 1 Department of Parasitology, Faculty of Medicine, University of Colombo, Kynsey Road, Colombo 8, Sri Lanka; 2 Wellcome Trust Centre for Human Genetics, University of Oxford, Oxford, UK; 3 Department of Chemistry, Faculty of Science, University of Colombo, 94, Kumaratunga Mawatha, Colombo 3, Sri Lanka

## Abstract

**Background:**

Antibodies against the merozoite surface protein 1_-19_ (MSP1_-19_) and the apical membrane antigen 1 (AMA1) of the malaria parasite (*Plasmodium vivax*) are proven to be important in protection against clinical disease. Differences in the production/maintenance of antibodies may be due to many factors including host genetics. This paper discusses the association of 4 anti-malarial antibodies with selected host genetic markers.

**Methods:**

Blood was collected from individuals (n = 242) with a history of malaria within past 15 years for DNA and serum. ELISA was carried out for serum to determine the concentration of anti-malarial antibodies MSP1_-19_ and AMA1 for both vivax and falciparum malaria. 170 SNPs related to malaria were genotyped. Associations between seropositivity, antibody levels and genetic, non-genetic factors were determined.

**Results:**

Age ranged 13–74 years (mean age = 40.21 years). Majority were females. Over 90% individuals possessed either one or more type(s) of anti-malarial antibodies. Five SNPs were significantly associated with seropositivity. One SNP was associated with MSP1_-19__Pv(rs739718); 4 SNPs with MSP1_-19__Pf (rs6874639, rs2706379, rs2706381 and rs2075820) and1 with AMA1_Pv (rs2075820). Eleven and 7 genotypes (out of 15) were significantly associated with either presence or absence of antibodies. Three SNPs were found to be significantly associated with the antibody levels viz. rs17411697 with MSP1_-19__Pv, rs2227491 with AMA1_Pv and rs229587 with AMA1_Pf. Linkage of the markers in the two groups was similar, but lower LOD scores were observed in seropositives compared to seronegatives.

**Discussion and conclusions:**

The study suggests that several SNPs in the human genome that exist in Sri Lankan populations are significantly associated with anti-malarial antibodies, either with generation and/or maintenance of antibodies for longer periods, which can be due to either individual polymorphisms or most probably a combined effect of the markers.

**Electronic supplementary material:**

The online version of this article (10.1186/s12936-018-2622-9) contains supplementary material, which is available to authorized users.

## Background

Malaria is considered as one of the most prevalent infectious diseases in the world with high morbidity and mortality. However, several countries plagued with this infection for centuries, were able to eliminate it from its borders between 2007 and present date, i.e. Armenia, Maldives, Morocco, Turkmenistan and United Arab Emirates [1].

Sri Lanka too joined the group of malaria–free countries in September 2016 when the World Health Organization (WHO) declared it a malaria-free nation [2]. Although the number of indigenous malaria cases remain as zero since October 2012 [3, 4], resurgence of malaria is possible through imported malaria cases that remains as a threat with 95, 49, 36, 41 and 57 cases of imported malaria reported in 2013, 2014, 2015, 2016 and 2017, respectively [5].

Naturally acquired antibodies play a major role in protection against malaria. Anti-parasite immunity is complex and stage specific. Following exposure to the parasite, both innate and adaptive immune responses are triggered. Antibodies produced against the parasite antigens have a protective role on subsequent infections, i.e. repeated infections trigger immunity to uncomplicated as well as severe malaria [6]. Furthermore, once acquired, anti-malarial antibodies are long-lasting [7] enhancing its importance in immunity to malaria infections.

The merozoite surface protein 1_-19_ (MSP1_-19_), which is a 195 kD protein on the surface of the merozoite, has been studied extensively as a vaccine candidate. Evidence from in vitro and challenge studies suggest that MSP1_-19_ might have an immunoprophylactic effect against malaria [8–10]. Furthermore, antibodies against both MSP1_-19_ of *Plasmodium falciparum* and *Plasmodium vivax* have been associated with protection against clinical disease in areas of stable transmission [11–14]. Similarly, the apical membrane antigen 1 (AMA1), which is essential in erythrocyte invasion by the parasite, has also been studied as a vaccine candidate [15] as well as an anti-malarial drug target [16]. AMA1 is also proven to be important in protection against clinical disease [13, 17, 18]. Thus, antibodies produced against these antigens are considered to play an important role in malaria immunity.

However, the role of antibodies as a protective armour against the infections may vary among individuals. This variability can account for the differences in susceptibility to malaria in individuals of similar age who live in malaria endemic areas. Differences in the production/maintenance of antibodies may be due to many factors including those related to socio-economic background, such as nutrition; epidemiology that include intensity of exposure; and host genetics.

The association between the host genes and antibody responses to malaria has been revealed by previous studies. A study done in Liberia in 1992 showed that antibody response against the malarial antigen Pf155/RESA is more consonant between monozygotic twins, when compared to dizygotic twins or age, sex matched sibling pairs [19]. Association between specific polymorphisms of genes and immune response has been documented by other investigators. Luoni et al. reported the association between the polymorphisms of the gene IL4-524 and anti-malaria antibody levels in a West African population [20]. In Sri Lanka, a study done in a previously malaria-endemic area revealed several single nucleotide polymorphisms associated with high antibody levels [7]. Afridi et al. reported the effect of polymorphisms of the genes HBB, IL-4, IL-12, TNF, LTA, NCR3 and FCGR2A on the levels of IgG responses against *P. falciparum* blood stage extracts suggesting the genetic effect on antibody response could be multifactorial [21]. A large multi-centre study conducted including 13,299 individuals from Africa and South Asia confirmed the fact that the genetic factors can determine an individual’s immune response to malaria [22].

This study looked into the relationship between humoral immunity against malaria (using 4 anti-malarial antibodies, i.e. MSP1_-19_ and AMA1 of *P. vivax* and *P. falciparum*) and host genetic polymorphisms of selected genes known to be associated with malaria in a previously endemic population of Sri Lanka.

## Methods

### Study site

The present study was conducted in 2 previously malaria endemic districts in Sri Lanka, i.e. Kurunegala district of North Western province and Moneragala district of Uva province. Four District Secretariat Divisions (DSDs) (out of 11 DSDs) from Moneragala district and 12 (out of 30 DSDs) from Kurunegala district were selected on the basis of presence of the District/Base hospitals or Medical Officer of Health (MOH) offices. Second stage clusters were determined after selecting the initial clusters. Two Grama Niladhari Divisions (GNDs) were randomly selected from each selected DSD by drawing lots (i.e. 8 and 24 GNDs from Moneragala and Kurunegala, respectively). Identification of locations were done within the secondary clusters by using relevant area maps from the Department of Census and Statistics of Sri Lanka [23].

### Recruitment of individuals

Voluntary participants from the selected area and people visiting either the area hospital or MOH offices for other blood investigations and who were willing to give a sample of blood for the present study were initially recruited for the study after obtaining informed consent. Before enrolment they were inquired on their history of malaria attacks, and people who have had malaria within past 15 years were recruited for the study. The selected individuals were given a unique identification number.

### Ethical considerations

Ethical clearance for this study was granted by the Ethics Review Committee of the Faculty of Medicine, University of Colombo, Sri Lanka (Certificate No: EC-11-191).

### Sample/data collection

Two millilitres of blood was drawn from the selected individuals by a trained nurse or a medical laboratory technician. This was divided into 2 tubes, one coated with EDTA (Ethylene Di-amine Tetra-acetic Acid) and the other a plain tube with each individual’s unique identification number. The samples in the EDTA tube were stored in a − 20 °C freezer until transfer to the laboratory in Colombo. Blood in the plain tube was allowed to clot for 4–5 h at 4 °C and was centrifuged at 12,000 rounds per minute (r.p.m.) to separate the serum. Serum was carefully extracted to a separate eppendorf tube labeled with the identification number. These serum samples were stored in the − 20 °C freezer until transfer to Colombo. Both the blood samples in EDTA and the serum samples were then transported to the Department of Parasitology, Faculty of Medicine, University of Colombo in ice packs in frozen condition. Data on age, sex and previous malaria history of individuals were collected.

### Enzyme-linked immuno-sorbent assay (ELISA)

A standard ELISA protocol described elsewhere [7, 24] was used to determine the seropositive and seronegative samples. In summary: anti-malarial antigens MSP1_-19_ (School of Life Sciences, Edinburgh, UK) and AMA1 (BPRC, The Netherlands), for both *P. vivax* and *P. falciparum*, were used in this protocol. ELISA plates (96 micro-well plates/Nunclon, Germany) were coated with 50 µL of antigen, i.e. MSP1_-19_-Pf, MSP1_-19_-Pv, AMA1_Pf or AMA1_Pv. Plates were incubated at 4 °C overnight and were washed three times with Phosphate Buffered Saline with 0.05% Tween 20 (PBS/T). To each well 100 µL of diluted serum samples (1: 200 for MSP1_-19_ and 1: 400 for AMA1 in 2% skimmed milk in PBS/T) were added. Each ELISA plate contained 46 test samples in duplicate, a known positive control in duplicate, a known negative control and a blank well. The plates were incubated at 4 °C overnight. They were washed 3 times in PBS/T and 50 µL of horse radish peroxidase-conjugated rabbit anti-human IgG (Sigma-Aldrich/Cat#A8792) diluted in 1:2000 in PBS/T was added to each well. This was incubated for 3 h at room temperature (27 °C–29 °C) before washing 3 times in PBS/T. To each well 50 µL of OPD substrate solution (Sigma-Aldrich/Cat#P1526)(0.4 mg/mL in solvent) was added. This was left for 15 min for the colour to develop. The reaction was stopped by adding 25 µL of 2 M H_2_SO_4_ per well. Then the plates were read at 492 nm using an ELISA micro-plate reader. The Optical Density (OD) value for each well was recorded separately.

Standard procedures were carried out throughout the laboratory tests to maintain the validity of the protocols. The same positive control with known concentration was included in every ELISA plate and a chart was maintained to identify any significant deviations (± 2 SD) of the OD value of the particular sample for normalization of the data.

### Formation of standard antibody concentration curves and determination of antibody levels

Standard antibody concentration curves were drawn using serum samples with known antibody concentration previously quantified using standard methods, at the Wellcome Trust Centre for Human Genetics, University of Oxford, UK. The corresponding OD values of the samples diluted 2-fold, 4-fold, 8-fold, 16-fold and 32-fold were obtained using the same ELISA protocol. The antibody levels in each sample were determined using the Microsoft Excel Trend function for linear regression. Individuals who obtained ≤ 0 antibody level value in regression were considered “individuals without antibodies (seronegatives)”—group A; and individuals with a value > 0 were considered as “individuals with antibodies (seropositives)”—group B.

### DNA extraction and genotyping

The blood collected in the EDTA coated tube was used for DNA extraction using QIAGEN QIamp DNA blood mini kit (Cat No: 51106) as per manufacturer’s instructions. Genotyping was done at the laboratory of the Wellcome Trust Centre for Human Genetics, University of Oxford UK. Hundred and seventy SNPs were selected on the basis of a review of reports of associations with malaria data from The MalariaGEN Consortium [25]. Genotyping of these selected SNPs (Additional file 1) were carried out by previously described methods [7]; in summary: five nanograms of gDNA was whole-genome amplified by primer-extension pre-amplification (PEP) using N15 primers (Sigma, UK) and Biotaq (Bioline, UK) polymerase as previously described by Zhang et al. [26]. Single nucleotide polymorphisms (SNPs) were assayed on the Sequenom^®^ iPLEX platform according to manufacturer’s instructions using diluted PEP DNA (1:10). Genotype calls were made using the Sequenom^®^ Typer v4.03 software [25].

### Data analysis

Data were entered to a Microsoft Excel spreadsheet and SPSS V19.0 spreadsheet. Association of antibody levels and non-genetic/genetic factors were determined. Presence/absence of antibodies (seropositivity) as well as the mean antibody levels between males/females and between the two districts were compared using Chi squared test/t-test, and the medians were compared using Mann–Whitney U test.

Hardy–Weinberg equilibrium (HWE) was assessed for all the SNPs using Haploview V4.2 software [27]. Genotypes were compared against presence or absence of antibodies (Chi squared test and binary logistic regression, adjusted for age, gender and location of residence); the mean antibody levels (ANOVA) and median antibody levels (Kruskal–Wallis test, Median test).

The study population was divided into 2 groups, i.e. individuals without any type of anti-malarial antibody (Group A) and individuals with one or more types of anti-malarial antibodies (Group B). Allele frequencies of all significant SNPs associated with the presence/absence of antibodies were calculated in these 2 groups separately. Minor allele frequencies of the two groups were compared by using the ratios of the two groups.

Haplotype analysis and determination of linkage disequilibrium (LD) was carried out using Haploview V 4.2 genetic data analysis software. Haplotype blocks were generated as described by Gabriel et al. in 2002, where a haplotype block was created if 95% of the informative comparisons are in strong LD [28].

When analysing the genotypes, individuals who had genotype mismatches and/or genotyping errors for a particular SNP were excluded. Thus, the number of individuals considered for each SNP was different. Three SNPs were removed from further analysis due to high rates of (> 10%) genotyping errors, i.e. rs5743810, hTNF-α376 and rs8176719. Antibody levels of 3 individuals could not be determined for errors generated obtaining OD values from ELISA. These individuals were excluded from analyses concerning antibody levels.

## Results

### Characteristics of the population

This study population comprised 242 individuals who confirmed they have had malaria within the past 15 years from 2 districts, i.e. Kurunegala (n = 136) and Moneragala (n = 106). The age ranged from 13 to 74 years with a mean age of 40.21 years. When individual sites (districts) were considered, ages ranged from 13 to 74 years in Kurunegala with a mean age of 36.77 years and a median of 32.50 years; and in Moneragala 16–70 years with a mean age of 44.64 years and a median of 46.50 years. Only 1.2% of the population was under 15 years of age, and the majority (> 75%) were between 16 and 60 years. There was no record of age in 5.4% (n = 13) participants (Table 1).

**Table 1:** Characteristics of the population

	N (%)
Age (years)	
> 15	3 (1.2)
16–30	79 (32.6)
31–45	57 (23.6)
46–60	55 (22.7)
> 60	35 (14.5)
Age not recorded	13 (5.4)
Gender	
Whole population	
Female	179 (74.0)
Male	61 (25.2)
Not recorded	2 (0.8)
Kurunegala	
Female	118 (86.8)
Male	17 (12.5)
Not recorded	1 (0.7)
Moneragala	
Female	61 (57.5)
Male	44 (41.5)
Not recorded	1 (0.9)

When the whole population was considered, the majority were females (74%), whereas when the districts were considered separately, 86.8% were females in the Kurunegala district, and 57.5% were females in the Moneragala district. The percentage of females in the Kurunegala district was significantly higher than the percentage of males when compared to the Moneragala district (χ^2^ = 81.18, *df* = 2, p < 0.05).

Within the study population 187 (77.3%) had AMA1_Pv antibodies, 185 (76.4%) had AMA1_Pf antibodies, 167 (69%) had MSP1_-19__Pv antibodies and 111 (45.9%) had MSP1_-19__Pf antibodies. Overall, only 23 individuals out of 242 (9.5%) did not possess any type of antibodies; Over 90% individuals of this study population possessed either one or more type(s) of anti-malarial antibodies tested.

### Association between non-genetic factors and presence/absence of antibodies (seropositivity)

The association of the presence of antibodies with age, gender and the location were tested. The population was divided into 5 age groups with equal intervals of 15 years to test the relationship between the age and presence of antibodies in individuals, i.e. 1–15 years (n = 3), 16–30 years (n = 79), 31–45 years (n = 57), 46–60 years (n = 55) and 61–75 years (n = 35). Anti-malarial antibody levels of MSP1_-19_ for both *P. falciparum* and *P. vivax* were significantly associated with age (χ^2^ = 14.90, *df* = 4, p = 0.005). Percentages of individuals with/without MSP1_-19__Pf were significantly different in age groups 2 (16–30 year olds) and 4 (46–60 year olds). In age group 2, the percentage of seronegatives (68.4%) was significantly higher than the percentage of seropositives (31.6%). In age group 4 the trend seen was the opposite with the percentage of seropositives (61.8%) being significantly higher than the percentage of seronegatives (38.2%) for MSP1_-19__Pf. For MSP1_-19__Pv the percentage of seropositives (54.5%) in age group 3 (31–45 year olds) was significantly higher than the percentage of seronegatives (45.5%) (χ^2^ = 9.6, *df* = 4, p = 0.048).

There were more seropositive males (for all 4 types of antibodies tested) when compared to seropositive females. A highly significant association could be observed between gender and presence of MSP1_-19__Pf antibody, with 72.1% of males being seropositive and majority (63.1%) of females being seronegative (χ^2^ = 22.78, *df* = 1, p < 0.05).

The percentage of seropositives was significantly higher for all tested antibodies in Moneragala district when compared to the Kurunegala district, except for anti-AMA1 antibodies for *P. falciparum* (Table 2).

**Table 2:** Percentages of individuals with and without antibodies in each district

Type of antibody	District	Percentage seropositives	Percentage seronegatives	Chi	p
MSP1_-19__Pf	Kurunegala	25.7	74.3	50.68	< 0.05
	Moneragala	71.7	28.3		
	Males	72.1	27.9	22.783	<0.05
	Females	36.9	63.1		
MSP1_-19__Pv	Kurunegala	55.1	44.9	27.89	< 0.05
	Moneragala	86.8	13.2		
	Males	77.0	23.0	2.622	0.105
	Females	65.9	34.1		
AMA1_Pf	Kurunegala	83.1	16.9	7.60	0.006
	Moneragala	67.9	32.1		
	Males	77.0	23.0	0.029	0.865
	Females	76.0	24.0		
AMA1_Pv	Kurunegala	68.4	31.6	13.97	< 0.05
	Moneragala	88.7	11.3		
	Males	85.2	14.8	3.085	0.079
	Females	74.3	25.7		

### Association between the non-genetic factors and the mean/median antibody levels

The association of antibody levels with age, gender and the residence were determined. When the whole population was considered, the mean antibody level increased with age, but decreased after ~ 60 years. A simple linear regression and curvilinear regression (including a quadratic model) was calculated to predict antibody levels based on age. There was no significant correlation (linear or non-linear) between age and antibody levels in the whole population or within age groups for any tested antibody.

Mean and median antibody levels were compared between the districts. The mean level of AMA1_Pf was significantly higher in Moneragala (mean = 6258.90 U/μL) when compared to Kurunegala (mean = 1927.91 U/μL) (t = − 2.703, *df* = 237, p = 0.007). Mann–Whitney U test was carried out to compare the median antibody levels between the two districts. The medians of all tested antibodies were significantly different between the districts (p < 0.05) (Additional file 2). The mean and median antibody levels between males and females were compared using the t test and Mann–Whitney U test. There were no significant differences in the means/medians of any tested antibodies between males and females except for median MSP1_-19__Pf antibody level, which was significantly higher in males (1296.55 IU/μL) when compared to females (0.00 IU/μL) (Mann–Whitney U test, p < 0.05).

Correlation between the antibodies was tested using the Pearson’s correlation. The greatest positive correlation was observed between the 2 vivax antibodies MSP1_-19_ and AMA1 and the least correlation was between MSP1_-19__Pf and AMA1_Pv. None of the tested antibodies were normally distributed within the study population or within each age group (Kolmogorov–Smirnov Test, p < 0.005) (Additional file 3).

### Association between the genotypes and the presence/absence of antibodies

The genotype distribution of the selected SNPs studied was in Hardy–Weinberg equilibrium (p > 0.05). Five SNPs were significantly associated with seropositivity in individuals (Table 3). Eleven and 7 genotypes (out of 15) were significantly associated with either the presence or absence of antibodies under the co dominant or dominant models, respectively. One SNP was associated with MSP1_-19__Pv (rs739718); 4 SNPs with MSP1_-19__Pf (rs6874639, rs2706379, rs2706381 and rs2075820) and1 with AMA1_Pv(rs2075820) (Table 3).

**Table 3:** Genotypes of the SNPs and the association with presence/absence of antibodies

Antibody type	SNP	Genotype	With antibody, N (%)	Without antibody, N (%)	Model	Comparison	OR (95% CI)	p-value
MSP1_-19__Pv	rs739718 (IL5)	TT	165 (70.2)	70 (29.8)	Co-dominant	TT/CT	5.893 (1.117–31.101)	0.019
		CT	2 (28.6)	5 (71.4)				
MSP1_-19__Pf	rs6874639 (C5ORF56)	AA	37 (39.4)	57 (60.6)	Co-dominant	AG/AA	2.054 (1.116–3.618)	0.12
		AG	60 (57.1)	45 (42.9)	Co-dominant	GG/AG	2.872 (1.339–6.159)	0.006
		GG	13 (31.7)	28 (68.3)	Dominant	AG/AA + GG	2.267 (1.364–3.816)	0.002
					Dominant	GG/AA +AG	0.488 (0.239–0.997)	0.046
	rs2706379 (C5ORF56)	CC	38 (38.4)	61 (61.6)	Co-dominant	CT/CC	2.203 (1.253–3.873)	0.006
		CT	59 (57.8)	43 (42.2)	Co-dominant	CT/TT	2.639 (1.213–5.738)	0.013
		TT	13 (34.2)	25 (65.8)	Dominant	CC/CT + TT	0.588 (0.349–0.993)	0.046
					Dominant	CT/CC + TT	2.314 (1.370–3.907)	0.002
	rs2706381 (C5ORF56)	CC	38 (38.4)	61 (61.6)	Co-dominant	CT/CC	2.165 (1.230–3.811)	0.007
		CT	58 (57.4)	43 (42.6)	Co-dominant	CT/TT	2.698 (1.244–5.849)	0.011
		TT	13 (33.3)	26 (66.7)	Dominant	CT/CC + TT	2.301 (1.362–3.888)	0.002
	rs2075820 (NOD1)	AA	29 (64.4)	16 (35.6)	Co-dominant	AA/AG	2.595 (1.261–5.341)	0.009
		AG	44 (41.1)	63 (58.9)	Co-dominant	AA/GG	2.665 (1.259–5.642)	0.009
		GG	34 (40.5)	50 (59.5)	Dominant	AA/AG + GG	2.626 (1.337–5.158)	0.004
AMA1_Pv	rs2075820 (NOD1)	AA	42 (93.3)	3 (6.7)	Co-dominant	AA/AG	4.725 (1.354–16.49)	0.009
		AG	80 (74.8)	27 (25.2)	Co-dominant	AA/GG	5.60 (1.583–19.80)	0.004
		GG	60 (71.4)	24 (28.6)	Dominant	AA/AG + GG	5.10 (1.514–17.17)	0.004

The minor allele frequencies of the significant SNPs of the two groups were calculated. The highest difference between the minor allele frequencies of the 2 groups, i.e. seropositive and seronegative, could be observed for the SNP rs739718 (1.669). The ratios for the other significant SNPs were within a close range (~ 1.1) except for rs2075820 in which the ratio was < 1 (Additional file 4).

### Association between the genotypes and the antibody level

Three SNPs were found to be significantly associated with the antibody levels of this study population viz. rs17411697 with MSP1_-19__Pv, rs2227491 with AMA1_Pv and rs229587 with AMA1_Pf (Table 4). The median antibody levels were significantly different among the individuals with different genotypes of these SNPs. Pair-wise comparison of the median antibody levels of individuals with different genotypes of rs17411697 revealed the median MSP1_-19__Pv antibody level of individuals with the genotype GT (median = 1116.42 IU/μL) was significantly higher when compared to the individuals with the genotype GG (median = 788.56 IU/μL) or genotype TT (median = 229.83 IU/μL) (median test, p = 0.042). For rs2227491 the median AMA1_Pv antibody levels of study subjects with CT genotype was significantly higher (median = 2113.65 IU/μL) when compared to the median antibody level of individuals with CC genotype (median = 1124.03 IU/μL) (p = 0.021). Similarly, the individuals with TT genotype of rs229587 had significantly higher AMA1_Pf antibody levels (median = 902.02 IU/μL) when compared to individuals with the genotype CC (median = 513.28 IU/μL) (p = 0.049).

**Table 4:** Association of genotypes with the median antibody levels: the median antibody levels of the individuals with each type of genotype of each SNP are indicated within brackets

SNP	Gene	Chrom. position	Associated antibody	Genotype (median antibody level IU/μL)	Genotype comparison	p
rs17411697	IL1A	2:113,259,694	MSP1_-19__Pv	GG (788.56)	GG v GT	0.815
				GT (1116.41)	GG v TT	0.057
				TT (229.83)	GT v TT	0.042
rs2227491	IL22	12:66,932,788	AMA1_Pv	CC (1124.03)	CC v CT	0.021
				CT (2113.65)	CC v TT	0.171
				TT (1783.59)	CT v TT	1.000
rs229587	SPTB	12:66,932,788	AMA1_Pf	CC (513.28)	CC v CT	1.000
				CT (592.27)	CC v TT	0.049
				TT (902.02)	CT v TT	0.124

Genotype group which the median antibody levels are compared and the p values of the pair-wise comparisons are stated

### Linkage disequilibrium and haplotype analysis

High linkage (D’ ~ 1.0 and r^2^ > 0.90) could be observed between 3 markers (i.e. rs6874639, rs2706379 and rs2706381) out of the 5 markers which were associated with seropositivity; but this could be observed in both seronegative (Group A) and seropositive groups (Group B). Linkage of the markers in the two groups were similar for but lower LOD scores (range 9.72–11.12) were observed in group A when compared to very high LOD scores (77.37–89.52) seen in group B. There was only one haplotype block containing rs2706379 and rs2706381 in both study groups. The 3 markers which were significantly associated with antibody levels were not in linkage with each other.

**Fig. 1:**
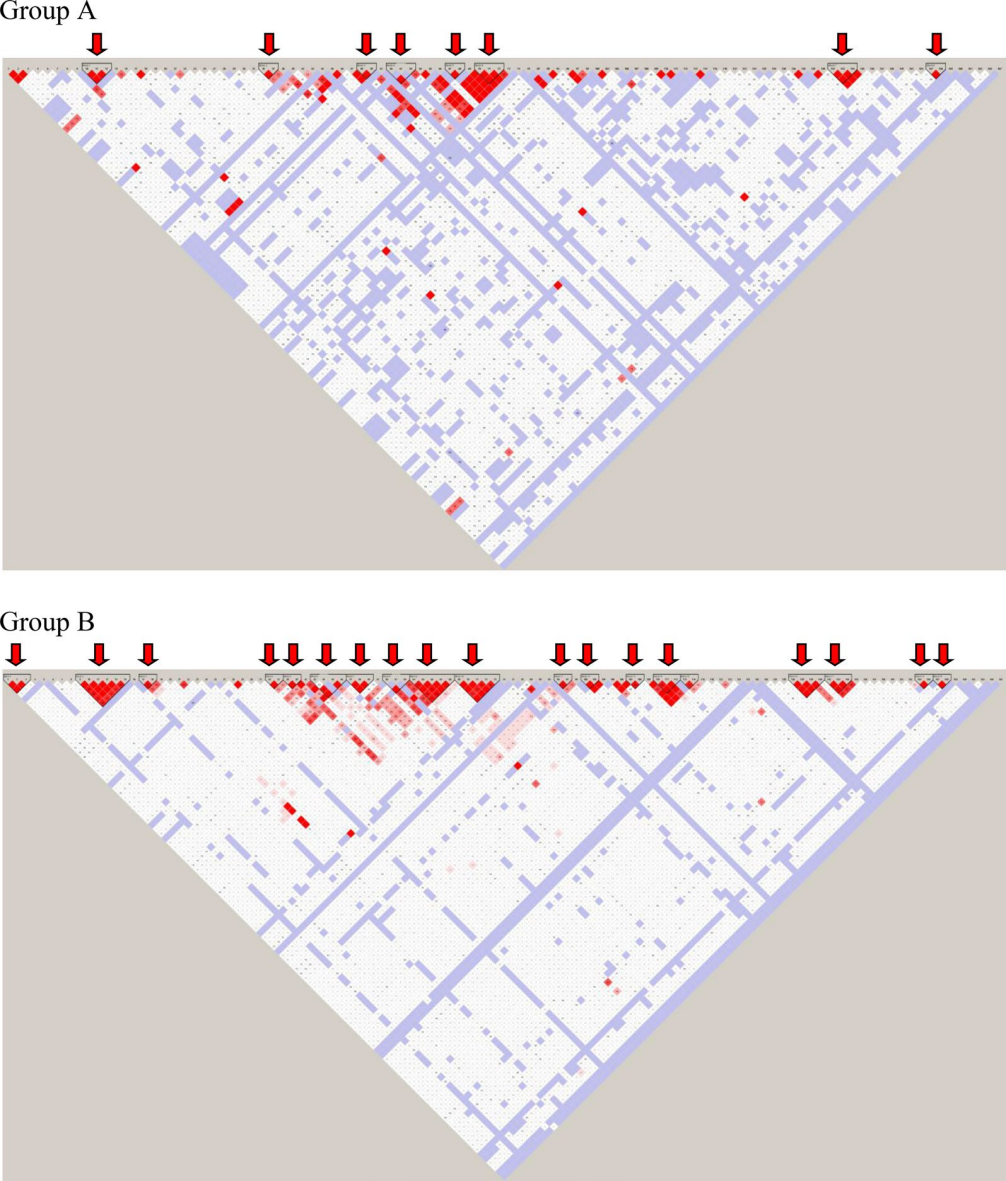
LD plots of the all the tested SNPs (n-170). The LD plots were generated separately for the two groups; group **a** sero-negative individuals and group **b** sero-positive individuals. The red arrows points where there is a block of SNPs (LD block) in high linkage disequilibrium. Red squares indicate high LD and the white square indicate very low or no LD

Eighteen blocks could be identified when all tested markers (n = 170) were considered in the LD plot generated for group B whereas there were only 8 blocks in group A. The 2 LD plots of the 2 groups displayed different patterns indicating differences in linkage of the tested SNPs between the two groups (Fig. 1).

## Discussion

This study was conducted to investigate the host genetic factors that may be associated with malaria immunity. Two hundred and forty-two individuals who verbally confirmed having had malaria within the past 15 years through recall memory from two previously malaria endemic districts of Sri Lanka were selected for this study. Only the individuals who provided convincing verbal evidence of having symptomatic disease of malaria in his/her past medical history were included in the study (out of 1186 interviewed only 242 were selected). The medical records were cross-checked where available. The majority was between 16 and 60 years of age and there were no children who were below 13 years. This could be easily explained since malaria transmission in Sri Lanka declined drastically approximately 15 years ago [3, 24] and the percentage of children below ~ 15 years with a history of malaria is extremely low. This very fact might be the reason for the observation that the seropositivity with regard to anti-malarial antibodies was significantly higher in older age groups (age group 3:MSP1_-19__Pv/age group 4: MSP1_-19__Pf) and lower in younger age groups (age group 2: MSP1_-19__Pf).

There was a progressive increase in antibody levels up to about 45–60 years after which there was a drop. The lower antibody levels in younger age groups most likely reflect the decreasing trend in malaria transmission seen in Sri Lanka since year 2000 and prior to total elimination [3] with an absence of exposure to malaria in the very young age groups; low levels of exposure of the age group 15–30 year olds; and higher cumulative exposure to the disease of the older age groups. In addition to the exposure, the older population tend to retain the antibodies, once acquired when compared to the younger individuals, which is in line with previous observations made elsewhere as well as locally [24, 29–31]. Ondigo et al. in 2014 reported the antibody MSP1_-19__Pf half-life being very short and sero-reversion being high for young children when compared to adults [31]. This observation is also supported by initial sero-epidemiological studies done by Warren et al. in the 1970s suggesting that antibodies in young children are often being short-lived and longer lasting in adults [30, 32]. Antibody secreting plasma cells are either short-lived or long-lived. In contrast to long-lived plasma cells, which survive and secrete antibodies for longer periods independently, short-lived plasma cells decline after an infection and are needed to be restored from memory B-cells [33, 34]. Akpogheneta et al. in 2008 suggested that the reason for short lived antibodies among children might be due to children having short lived plasma cells, which decline rapidly after an infection when compared to adults who can produce long-lived plasma cells derived from the germinal centre [6].

The relationship between age and immunity has been documented by others. Reily et al. described that the antibody prevalence of MSP1_-19_ of *P. falciparum* increased with age in a cross sectional survey done in Gambia [14]; and Noland et al. made the same observations with several types of antibodies against *P. falciparum* malaria parasites including AMA1 and MSP1_-19_ in a population in Western Kenya [35].

The frequency of seropositivity in males was much higher when compared to that in females. Similarly, the mean and median levels of antibodies of male individuals were higher (ranged between 71 and 86%). The percentage of females with anti-malarial antibodies ranged between 60 and 75% for all the tested antibodies except for MSP1_-19__Pf (only 36.9% of females were with MSP1_-19__Pf antibody) making the percentage of females with the antibody significantly lower when compared to the percentage of males with this antibody. This could be due to the differences in the level of exposure of males and females to mosquito bites, as a result of the differences in clothing patterns between the sexes. In rural areas, men rarely cover the upper parts of their bodies, and they also engage in outdoor activities more, including “*Chena*” cultivation, which takes place in the midst of jungle areas. Therefore, men have a higher chance of exposure to the disease, over women who mainly engage in house hold activities away from insect-infested areas outdoors. Males have a high tendency of being exposed to the infection because of the reasons discussed above and could account for this observation. Ondigo et al. in 2014 reported that antibodies to *P. falciparum* antigens may vary by time since last exposure [31]. Furthermore, antibodies to MSP1_-19_ have long half lives once acquired in older age (compared to the younger age groups) [29, 35, 36]. The mean age of females in this study was 39.24 years, which was less than the mean age of the males (43.59 years) indicating that the female group is consisted of much younger individuals when compared to the male group. Thus, there is a higher chance for MSP1_-19_ to persist in males rather than in females, but in contrast, a similar percentage of seropositive males (77%) and females (76%) were observed for anti-AMA1_Pf. It is documented that the age has a lesser effect on the half life of anti-AMA1_Pf when compared to anti-MSP1_-19__Pf [29], which might explain the compatible levels of seropositivity in AMA1_Pf between the sexes.

Differences in gender with regard to malaria immunity, malaria susceptibility and epidemiology have been documented by other investigators as well. Mendis et al. in 1990 made the same observation in an endemic population in Sri Lanka, where the incidence rate of malaria was significantly higher in males than females, especially in individuals aged 16–56 years. They concluded that the exposure to the disease among adults might be associated with occupational related movements, which further supports the observations made in this study [37]. In India, effects of exposure to the disease upon males were larger than those for females [38]. However, Giha et al. described that the effect upon gender may vary according to the season, locality, and other demographic characteristics of the populations in question [39].

Interestingly, the seropositivity for AMA1-Pf was higher when compared to that of MSP1_-19__Pf in the female subpopulation within the study group, which is more apparent in Kurunegala in the district-level analysis. AMA1-Pf is apparently known to be highly immunogenic when compared to the MSP1_-19_ antigen of the *P. falciparum* parasite [29, 40]. Weiss et al. reported that increase of MSP1_-19_ specific memory B cells in *P. falciparum* infected individuals with age is lower than that of increase of AMA1 specific memory B cells, suggestive of an outcome based on repetition of exposure rather than a function of age [41]. Furthermore, Badu et al. in 2012 reported that highly immunogenic AMA1_Pf tend to saturate into detectable levels (by ELISA) even in low/moderate transmission settings compared to MSP1_-19__Pf levels which needs repeated exposure for saturation [42]. Such reasons may explain the low MSP1_-19__Pf seropositivity when compared to that of AMA1_Pf of the females, due to their presumed limited exposure to malaria based on behavioural habits. The predominantly female population within the study group in Kurunegala district (i.e. 86.8%) is likely to have influenced the overall seropositivity rates calculated for the district against each *P. falciparum* antigen. In contrast the percentage of females in the study population from Moneragala is relatively low (57.5%), hence has a lesser effect upon total seropositivity calculated for the district.

Furthermore, the differences in socio-demographic characteristics of the 2 populations could have an effect upon the differences of seropositivity seen between the 2 districts, where Kurunegala being a relatively more urban area, where a considerable proportion of the population lives near the town with lesser exposure to the disease. In contrast, Moneragala district is more a rural area, with males engaged in “*Chena*” cultivation amidst jungles exposed to higher risk of the disease. Therefore, the population of Moneragala district have a higher chance to repeated exposure of malaria leading to higher seropositivity rates for MSP1_-19__Pf, compared to the population from Kurunegala district, where people with lesser exposure will indicate a higher proportion of seropositivity for AMA1_Pf (due to high immunogenicity), but not for MSP1_-19__Pf. In addition, the differences in sub class, half life and the polymorphisms of the antibodies could have an effect upon this observation [42].

The seropositivity rates as well as the mean/median levels of antibodies were significantly higher in 3 out of 4 tested antibodies in the Moneragala district, when compared to those from the Kurunegala district. Both Moneragala and Kurunegala were considered as high malaria endemic areas 10 years ago when the burden of malaria was high (before the year 2000). Moneragala district is considered as a rural area with an annual parasitic incidence (API) of 0.1–0.9 in year 2010, when compared to Kurunegala district, which had an API of 0.01–0.09 during the same period [43]. This most probably, explains the higher seropositivity rates as well as the high means and medians of the tested antibodies in the Moneragala district when compared to Kurunegala district.

The highest correlation observed among the antibodies was between those against *P. vivax* antigens. This might be explained using malaria transmission dynamics that prevailed in Sri Lanka in the past, with *P. vivax* being the predominant causative agent for malaria in Sri Lanka, which may have an indirect effect on correlation, as the number of individuals who were infected with *P. falciparum* might be less compared to *P. vivax.* The correlation between antibodies is likely to be affected by the level of infection and also the age [22].

Five single nucleotide polymorphisms (SNPs) out of the selected host SNPs studied were associated with either the presence or absence of antibodies, and 3 SNPs with high antibody levels. These SNPs (or genes that contain them) have been previously implicated to be associated with immunity, disease severity and/or protection against malaria [44, 45]. Three of the SNPs, which were associated with the presence/absence of the antibodies (viz. seropositivity), were located in the C5ORF56 gene, and 1 SNP in IL5 gene. Two SNPs in the C5ORF56 gene (i.e. rs6874639 and rs2706381) and rs739718 in IL5 gene are intron-variants. Intron-variants are generally considered as variants that do not directly have an impact on gene regulation, but there is a possibility that they can enhance gene expression or affect splicing of mRNA [46, 47]. Greenwood et al. in 2003 reported of introns containing enhancer elements that are capable of increasing gene expression by 2-folds in human dopamine transfer gene [48]. In a recent study, an intron variant which is in HPSE gene was identified as a variant that is associated with SNP—dependent enhancer activity especially in malignancies [49]. Maiga et al. in 2013 also supports this observation where they reported the association between the same SNP (rs739718) and falciparum antibodies AMA1 and MSP1_-19_ [44]. On the other hand, the intron variants can be linked with another SNP that actually has an impact on the regulation of the gene.

The SNP rs2706379 overlaps 4 genes (i.e. C5ORF56, RF00019, AC116366.1 and C5ORF56AC116366.3), and 11 transcripts [50]. The major possible consequences of this SNP are listed as an intron variant (44%) and non-coding transcript variant (25%) and to a lesser extent as a missense variant within an exon which has the possibility of making a protein/amino acid change. The major impact of this SNP is stated as a “modifier” [50]. The importance of non-coding DNA in fundamental gene processes, i.e. gene regulation and 3D chromatin folding has been documented which in turn can affect gene regulation and gene expression [51].

Since the relationship of the gene C5ORF56 (previously known as LOC441108) with malaria susceptibility has been described before [52], rs2706379 was considered as a part of this gene. The base change of this variant is C>T, and in the case of acting as a missense variant the possible amino acid change is Ser>Pro. In this population the heterozygous (CT) individuals were significantly associated with seropositivity when compared to the homozygous individuals. Therefore, it could be speculated that these polymorphisms in this SNP might have an effect upon gene regulation, which might be important for immune responses. On the contrary, the SNP could also have an effect upon malaria susceptibility and/or resistance, which intern could affect the seropositivity.

Both C5ORF56 and IL5 genes are included in the 5q31–33 cytokine gene cluster. The 5q31–33 region contains genes that encode the T helper 2 type cytokines or cytokine receptors and these genes are considered as strong candidates for determining of severity of malaria [52]. Furthermore, Garcia et al. in 1998 and Flori et al. in 2003 suggested that genes located on chromosome 5q31–33 are important in the control of malaria blood infection levels [53, 54]. IL5 levels were also observed to be significantly high in mild malaria patients when compared to severe malaria or cerebral malaria patients who had low/intermediate levels of IL5 [55]. IL5 is expressed by eosinophils [56]. Eosinophils are responsible for the elimination of antibody bound parasites by releasing cytotoxic granule proteins [57] indicating the importance of polymorphisms of the IL5 gene in malaria infections.

NOD (Nucleotide binding Oligomerization Domain) proteins involving Nod like receptors have been identified as key host molecules in innate immune and inflammatory responses. Once stimulated by pathogens they activate the signaling pathways leading to activation of pro-inflammatory cytokines and chemokines [58]. Furthermore, up-regulation of Nod proteins upon exposure of peripheral blood mononuclear cells (PBMCs) to malaria sporozoites has been observed [59] and influence of Nod proteins on cytokine levels involving malaria pathogenesis, i.e. IFN-γ [60] supports the theory that alludes to the importance of genes that encode Nod Protein in malaria. The SNP rs2075820 in the NOD1 gene was previously reported as a polymorphism associated with clinical malaria in Mali [44]. In this study, it was found that rs2075820 (located in the NOD1 gene) is associated with seropositivity to both MSP1_-19__Pf and AMA1_Pv. This particular SNP is a missense variant (C>T) with an ability of changing the amino acid by changing the transcription codon from **G**AG (Glu) to **A**AG (Lys), indicating that polymorphisms at this particular chromosomal position could alter the structure/function of the NOD protein, which ultimately might have an influence over immune responses to malaria. The SIFT score and the PolyPhen score reflects whether an amino acid substitution affects protein function. This variant however, has a SIFT score of 0.05 (amino acid change marginally tolerant) and a PolyPhen score of 0.266 (amino acid change tolerant) which indicates that the amino acid change due to the polymorphism has a limited effect upon the structure and/or function of the protein [50].

The SNP rs17411697, which is a missense variant (340 G>T), and is capable of altering the amino acid sequence (Ala>Ser) which has a SIFT score of 0.02 and a PolyPhen score of 0.637 indicating this amino acid change is deleterious and has a significant effect upon the protein In this study, it was evident that the heterozygotes of this SNP were significantly associated with higher levels of MSP1_-19__Pv antibodies. A study done in Tanzania revealed that carriage of the T allele of the rs17411697 was associated with an increase risk of acidosis in severe malaria [45]. This marker is located in the IL1A gene, which encodes IL1 pyrogen. Walley et al. in 2004, who found significant associations between the variants of IL1A gene and severe malaria suggested that IL1 may have a role to play in inflammatory damage due to malaria parasites in the brain in cerebral malaria; thus concluding that variants of the IL1A gene may also be associated with cerebral malaria [61].

The SNP rs2227491 located in the IL22 gene was found to be associated with lower levels of AMA1_Pv antibodies with the homozygote individuals carrying the mutant allele (C) having significantly lower antibody levels. IL22 is produced mainly by activated Th1 cells and enhances the innate immunity, which activates a series of non-specific defense mechanisms immediately after a pathogen enters the body. It can be hypothesized that the polymorphisms in this marker may have significant effect on IL22 production, thus positively influencing the innate immune system with a resultant protective role against malaria infection in the host. Thus, individuals with the mutant allele (C) may have lower antibody levels due to their inherent protection against malaria infection. However, opposing views also have been expressed by others with the mutant allele (C) of this marker identified in association with susceptibility to malaria and the ancestral allele (T) in association with protection against severe disease [62]. Thus it can also be argued that the individuals with the mutant C allele tend to produce low levels of antibodies and might be more susceptible to malaria compared to the individuals carrying the ancestral T allele. Furthermore, since rs2227491 is an intron-variant the effect of this marker on antibody levels might also be due to linkage as well. Therefore, more advanced studies with serological, epidemiological and genetic data will be needed to make exact inferences on the effect of these alleles on malaria.

The SNP rs229587 had a marginally significant association with lower levels of AMA1_Pf antibodies. This marker is also an intron-variant, thus this effect upon the antibody levels could be due to other markers, which are in linkage with rs229587.

The main limitation of this study is the low number of study participants (n = 242) and low number of SNPs (n = 166) included, compared to the whole genome studies with larger populations, which adds more power to the genetic association analysis. However, the sample numbers and the number of SNPs studies had to be limited due to logistical reasons that prevailed during that time.

The majority of the study population was females. This might have an effect upon the results as significant associations between gender and antibody levels were observed within this population. However, appropriate statistical measures were taken to minimize these effects.

It is also noteworthy that a possible bias may exist towards the genetic analyses used in this study since the genetic variants explored are listed as candidate SNPs, which are associated with either severe and complicated or uncomplicated malaria and has been selected after extensive review of published data from MalariaGEN consortium [25]. Thus there is a possibility that these SNPs may exhibit an association with antibody levels or seropositivity, when actually the association of these tested SNPs might be with other aspects of malaria (e.g. disease severity). Although advanced statistical methods are available to minimize the effects of other confounding variables, absence of related data (such as data on parasitaemia, fever scores, disease severity) is a limitation.

## Conclusion

The study suggests that several SNPs in the human genome that exist in Sri Lankan populations are significantly associated with anti-malarial antibodies, either with generation and/or maintenance of antibodies for longer periods, which can be due to either individual polymorphisms or most probably a combined effect of the markers. Further studies with larger study population size will be needed for confirmation of these observations. The levels of anti-malarial antibodies seem to be a result of repeated exposure to the disease, and influenced by age, gender and the malaria transmission pattern of the location.

## Additional files

**Additional file 1.** List of Single Nucleotide Polymorphisms [SNPs] genotyped.

**Additional file 2.** Comparison of median antibody levels between the districts.

**Additional file 3.** Frequency distribution of the antibodies in the study population and in each age group for the tested antibodies.

**Additional file 4.** Minor allele frequencies of the SNPs significantly associated with presence of antibodies.
