# Supplementary material for: Host genetic polymorphisms and serological response against malaria in a selected population in Sri Lanka
Source: Malar J. 2018 Dec 17;17:473. doi: 10.1186/s12936-018-2622-9 (PMC6296029; doi:10.1186/s12936-018-2622-9)
Supplement: Supplementary file 2 — Additional file 2. Comparison of median antibody levels between the districts. [file 12936_2018_2622_MOESM2_ESM.docx]

Additional file 2:

Comparison of median antibody levels between the districts

|  | Median antibody level | | p |
| --- | --- | --- | --- |
|  | Kurunegala | Moneragala |  |
| MSP1_Pv | 256.17 | 1302.03 | 0.000 |
| MSP1_Pf | 0.00 | 973.34 | 0.000 |
| AMA1_Pv | 1644.73 | 1933.35 | 0.000 |
| AMA1_Pf | 986.64 | 295.53 | 0.002 |
