# Supplementary material for: Host genetic polymorphisms and serological response against malaria in a selected population in Sri Lanka
Source: Malar J. 2018 Dec 17;17:473. doi: 10.1186/s12936-018-2622-9 (PMC6296029; doi:10.1186/s12936-018-2622-9)
Supplement: Supplementary file 3 — Additional file 3. Frequency distribution of the antibodies in the study population and in each age group for the tested antibodies. [file 12936_2018_2622_MOESM3_ESM.docx]

Additional file 3: Frequency distribution of the antibodies in the study population and in each age group for the tested antibodies.

|  |  |
| --- | --- |
|  |  |

| Age group | AMA1_Pf | AMA1_Pv | MSP1_Pf | MSP1_Pv |
| --- | --- | --- | --- | --- |
| 1 | **** | **** |  | **** |
| 2 | **** | **** | **** | **** |
| 3 | **** | **** | **** | **** |
| 4 | **** | **** | **** | **** |
| 5 | **** | **** | **** | **** |
