# Supplementary material for: Host genetic polymorphisms and serological response against malaria in a selected population in Sri Lanka
Source: Malar J. 2018 Dec 17;17:473. doi: 10.1186/s12936-018-2622-9 (PMC6296029; doi:10.1186/s12936-018-2622-9)
Supplement: Supplementary file 4 — Additional file 4. Minor allele frequencies of the SNPs significantly associated with presence of antibodies. [file 12936_2018_2622_MOESM4_ESM.docx]

Additional file 4:

Minor allele frequencies of the SNPs significantly associated with presence of antibodies. The ratios of the minor allele frequencies of group A (Sero – negative individuals)/ group B (Sero – positive individuals) which indicate the distance or the closeness of MAFs of the two groups is also indicated.

|  | Minor Allele Frequency (MAF) | |  |
| --- | --- | --- | --- |
| SNP | Group A | Group B | Ratio |
| rs6874639 | G (43.5) | G (38.7) | 1.124031 |
| rs2706379 | T (41.3) | T (37.0) | 1.116216 |
| rs2706381 | T (41.3) | T (37.3) | 1.107239 |
| rs739718 | C (2.17) | C (1.3) | 1.669231 |
| rs2075820 | A (28.3) | A (43.3) | 0.65358 |
